# Risk-taking, loss aversion, and performance feedback in dynamic and heterogeneous tournaments

**DOI:** 10.3389/fpsyg.2023.1223369

**Published:** 2023-11-09

**Authors:** Qing Yang, Yangqing Zhao

**Affiliations:** ^1^School of Physical Education, Soochow University, Suzhou, Jiangsu, China; ^2^School of Physical Education and Health, Wenzhou University, Wenzhou, Zhejiang, China

**Keywords:** loss aversion, risk-taking, performance feedback, slack, decision making

## Abstract

Within the context of professional football, we examined the impact of the interim game state on risk-taking and performance during a dynamic tournament. This study used 9,256 segments from the top five European football leagues as samples. These segments were derived from 1,826 games played during the 2017–2018 season. Poisson regression was employed to analyze the distinct effects of game state and heterogeneity on performance under pressure. The results indicated that stronger teams tended to increase their attack intensity when facing weaker opponents. However, as their lead expanded, they tended to reduce their attack intensity, particularly in matches with heterogeneous characteristics. Moreover, teams trailing in scores tended to intensify their attacks but achieved little. However, leading teams consistently underperformed in terms of blocked shots and corner kicks. Additionally, tied teams systematically exhibited lower performance in shots on target and free kicks compared to leading teams, despite having a higher motivation to excel. These findings extend our understanding of how risk-taking and performance depend on disclosing information regarding relative performance.

## 1. Introduction

Most economic activities and sports competitions are held as tournaments. Individuals or teams competing in tournaments are compensated according to their relative, rather than absolute, performance. Tournaments influence individual behaviors such as risk-taking and performance under stress (Genakos and Pagliero, 2012). Additionally, because tournaments are typically dynamic in nature, it is crucial to understand how the disclosure of information regarding relative performance during a competition can impact players’ subsequent behavior. Moreover, tournaments are characterized by relative performance evaluations that reward the better or best performer with the largest prize. Therefore, it is essential to comprehend how the unveiling of information on relative performance during a competition may affect players’ subsequent behavior. In light of tournament theory (Lazear and Rosen, 1981), several studies highlight the role of tournaments in reorganizing information during a game that reveals heterogeneity (interim rank or intermediate scores and quality of the opponent). There is evidence on risk-taking and loss aversion in heterogeneous tournaments.

The tournament literature suggests that participant heterogeneity and perceptions of unfairness can make tournaments less effective. A heterogeneous underdog quickly realizes their minimal chance of winning and reduces their effort level, which secures the achievable rank. Conversely, the favorite anticipates a noticeable competitive edge early on.

According to tournament literature, when competing, the more effort you put in, the higher your chances of winning.

### 1.1. Risk-taking

Risk-taking is a mindset that embraces uncertainty and seeks opportunities for growth and achievement. Numerous studies have shown that losing teams exhibit more offensive aggression, such as making risky substitutions (Grund and Gürtler, 2005) and increasing the number of three-point shots (Grund et al., 2013). Similarly, risk-taking is reinforced by a higher expected benefit (Lee, 2004). More significant risks, such as higher announced weights in weightlifting competitions, are taken by bottom-ranked players or individuals trailing just behind interim leaders (Genakos and Pagliero, 2012; Neuberg and Thiem, 2022). Similarly, individuals are more reckless in a race when overtaken by lower-ranked counterparts (Becker and Huselid, 1992; Bothner et al., 2007). Pressure from losing motivates teams to improve their performance. For example, basketball teams trailing by one point in the first half are more likely to win a game than teams leading by one point in the first half (Berger and Pope, 2011).

### 1.2. Loss aversion

Loss aversion, as proposed by Tversky and Kahneman (1979), suggests that in tournaments people do not assign equal value to gains and losses. Consequently, losses are given significantly more weight than gains. The effects of expected revenues and losses are deeply unbalanced. In the context of sports, several studies have suggested that individuals and teams have the stimulus to compete more defensively when in the lead. Romer (2006) showed that head coaches in the National Football League are highly risk-averse regarding their fourth-down strategies. Coaches elect to punt or kick a field goal in many fourth downs, despite solid evidence suggesting that these decisions are economically inefficient. Pope and Schweitzer (2011) found that professional golfers underperform in hitting birdie putts when leading while hitting more accurately during lagging. Similarly, Elmore and Urbaczewski (2021) found evidence of significant loss-averse behavior among the world’s best golfers based solely on par ratings. Genakos and Pagliero (2012) demonstrated that competitors methodically outperform their rivals when they are ranked closer to the top, even though they already have a higher incentive to perform well.

In a soccer setting, Schneemann and Deutscher (2017) found that players run more when they are one goal ahead and lower their effort when they are trailing. Loss aversion drives all these behaviors as players tend to attach more value to potential losses than for potential gains and adjust their efforts accordingly.

### 1.3. Heterogeneity

Unequal tournaments, where one participant is initially stronger than the others, lead to less effort from the contestants. The greater the initial disadvantage of the underdog, the more effort they need to exert in order to compensate for the handicap. Conversely, the favorite is aware of their advantages and can reduce their efforts without jeopardizing their chances of winning (Berger and Nieken, 2016). The idea that performance incentives such as prizes can motivate individuals is supported by empirical evidence, as demonstrated by studies on golf tournaments in 1990 (Ehrenberg and Bognanno, 1990).

### 1.4. Home advantage and the crowd effects

Home advantage can be defined as the phenomenon in which home teams in sports win more games played in a balanced home-and-away schedule. Home advantage plays an important role in association football and its existence is well documented (Pollard, 1986).

Several studies on home advantage have focused on the role of crowds. This study has explored various ways in which the crowd may directly or indirectly affect the game and has found evidence of home advantage resulting from crowd-induced officiating influences (Nevill et al., 2002; Dohmen, 2008) and the presentation of the crowd (Nevill et al., 1996; Dohmen, 2008), which can impact the outcome of the game.

There is evidence that when playing at home, the crowd can influence the referees’ decisions, giving the home team an advantage.

In summary, numerous empirical studies have documented that asymmetric abilities and interim game states influence player behavior. There is less evidence on the incentive effect of the interaction between the two on risk-taking or loss aversion. This study contributes to the literature by examining how professional male soccer players react to i) the interim game state of leading or trailing, ii) performance feedback on the heterogeneity of contestants, and iii) effect of the interaction between these two factors.

This paper is organized as follows. First, we provide a brief overview of the data structure and model specifications. Next, we present the primary findings. Third, we examine the empirical approach and conclude with remarks.

## 2. Materials and methods

### 2.1. Samples

We examined the information gathered by Wyscout on 3,071,395 events from 1,826 matches played during the 2017–2018 season in the top five European football leagues, namely, the English Premier League, Spanish La Liga, Italian Serie A, German Bundesliga, and French Ligue 1. The data contained all the spatiotemporal events (passes, shots, fouls, etc.) that occurred during each match for an entire season of five prominent soccer competitions. Each match contained information regarding its position, time, outcome, players, and characteristics. Furthermore, the datasets are accessible to the public (Pappalardo et al., 2019).

### 2.2. Variables

#### 2.2.1. Dependent variables

Risk-taking. Risk-taking was quantified as the team’s offensive intensity per segment. The team’s offensive intensity included shots on target, shots off target, blocked shots, free kicks, and corner kicks. In this study, we focused on segments as the central elements of our conceptual narrative. These segments were categorized based on the game state and match location. For the analysis, we utilized various dependent variables, including the number of shots on target (ShotOn), shots off target (ShotOff), blocked shots (ShotBlocked), free kicks (Freekick), and corner kicks (Cornerkick) conducted by each team within a given segment.

#### 2.2.2. Independent variables

**Figure 1:**
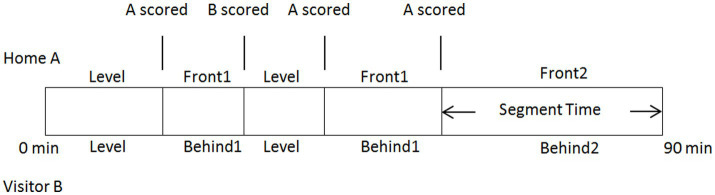
Visual depiction of the procedure for dividing data into segments.

Intermediate information. Figure 1 presents intermediate information regarding the dynamic state of the match, which is determined by the game state and segment. The game state is defined as the current goal difference in the match, whereas the segment corresponds to different game states. Specifically, the game state is categorized into five groups: “draw” (used as the reference category), “one goal behind,” “two or more goals behind,” “one goal lead,” and “two or more goals lead.”

Heterogeneity effect. To gauge heterogeneity, we derived the salary ratio from the Global Sports Salaries Survey (2017),^1^ a comprehensive repository of salary data across all leagues in our dataset. The salary ratio, which represents the ratio of a team’s salary to that of its opponent, was used as a proxy measure. According to Zhao and Zhang (2021), a higher salary ratio indicates the team’s greater ability relative to its opponent.

Venue. A dummy variable was used to indicate the venue of the match, with a value of 0 assigned to the away team and a value of 1 assigned to the home team.

#### 2.2.3. Control variables

The variable elapsed time (segment duration) captured the second of regular playing time.

### 2.3. Statistical analysis

**Figure 2:**
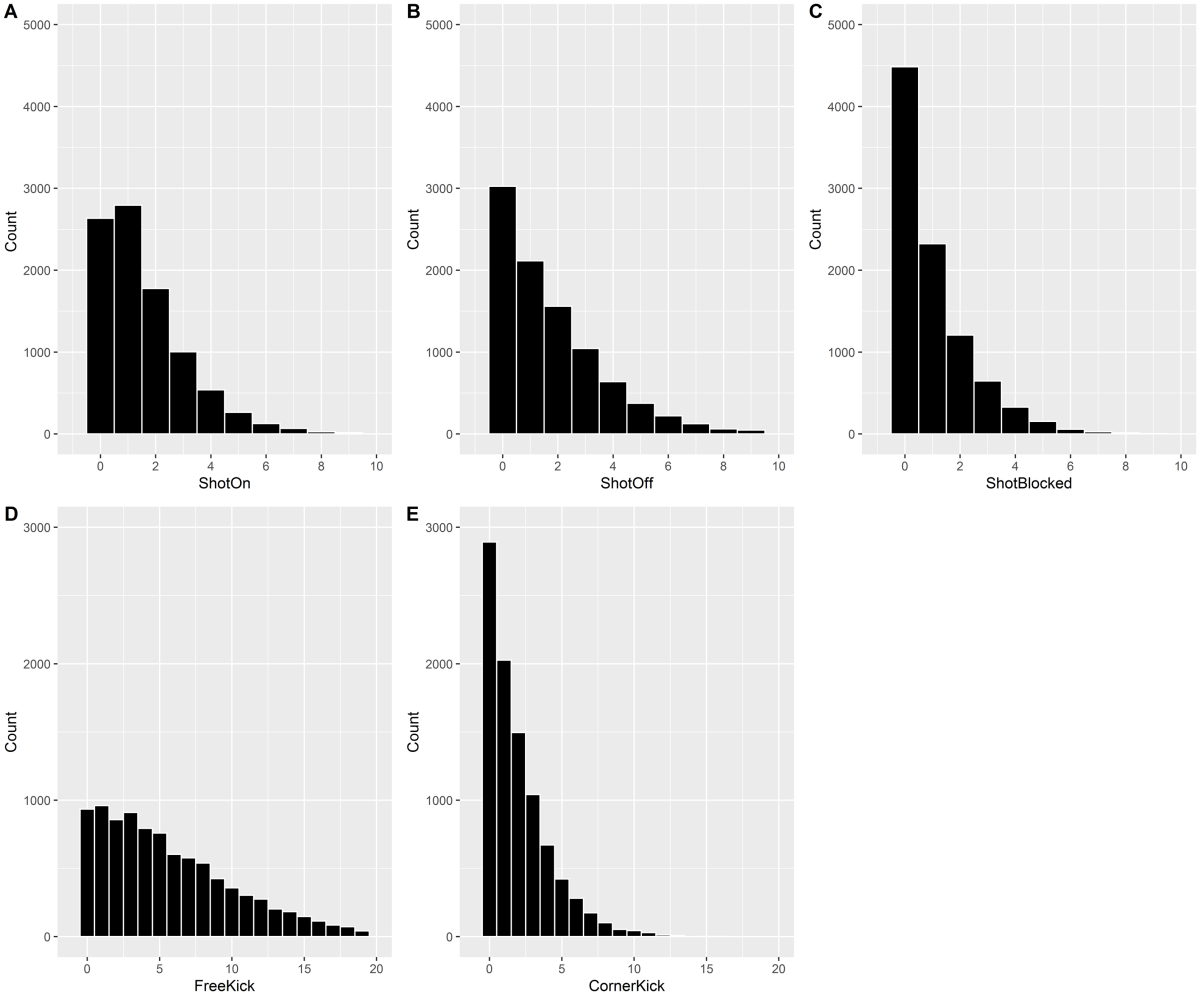
Histogram of ShotOn **(A)**, ShotOff **(B)**, ShotBlocked **(C)**, Freekick **(D)**, and Cornerkick **(E)** counts.

As shown in Figure 2, a thorough examination of the data revealed that neither homoscedasticity nor normality assumptions were met under any circumstances. As a result, generalized linear models (GLMs) were used. All the dependent variables were classified as count data with no upper limits. In terms of the five dependent variables, the residual deviance and Pearson χ^2^ both exceeded one time the residual degrees of freedom, with all overdispersion parameters being approximately 1 (ranging from 0.993 to 1.325). Poisson regression worked for our count data because of the underlying assumption that the variance was equal to the mean of the data (Zuur et al., 2009, p. 3).

Consequently, it is reasonable to assume that there was no overdispersion, and a GLM analysis (using the Poisson family) was performed to validate the model (Zuur et al., 2009, p. 196).

Therefore, the numbers of shots on target (ShotOn), shots off target (ShotOff), blocked shots (ShotBlocked), free kicks (Freekick), and corner kicks (Cornerkick) conducted by a team in one segment were used as the dependent variables in the GLM models. Moreover, GameState, venue, salary ratio, live attendance, and elapsed time were used as the explanatory variables.

In this study, pairwise comparisons were made to separate the means in all GLM analyzes (performed using the “multicomp” package developed by Hothorn et al. (2012)), and statistical analyzes and graphs were created with R 4.0 (developed by the R development core team in 2018). The “lmerTest” package was used to estimate all the models, the “Rsq” package [developed by Zhang (2016)] provided R^2^ values for the models, and the “Gmodels” package [developed by Warnes et al. (2009)] provided confidence intervals for the models.

Taking shots on target as an example, $Y_{i}$ , the number of shots on target committed in segment *i*, is Poisson distributed with mean $\mu_{i}$ . $LAttendance_{i}\;$ is the live attendance in segment *i*. The Poisson GLM for the shots-on-target data is given by the following equations:


$$Y_{i}\sim Poisson\left(\mu_{i}\right)\;and\;E\left(Y_{i}\right)=\mu_{i}\;and\;\mathrm{var}\left(Y_{i}\right)=\mu_{i},$$

$$\begin{array}{l} \log\left(\mu_{i}\right)=\alpha +\beta_{0}\times {\mathrm{GameState}}_{i}+\beta_{1}\times {\mathrm{Venue}}_{i}\\ +\beta_{2}\times {\mathrm{ElapsedTime}}_{i}+\beta_{3}\times \mathrm{Salary}\_{\mathrm{ratio}}_{i}\\ +\beta_{4}\times LAttendance_{i}+\beta_{5}\times {\mathrm{GameState}}_{i}*\mathrm{Salary}\_{\mathrm{ratio}}_{i}. \end{array}$$

## 3. Results

### 3.1. Game state effect

Table 1 displays the results of the Wald chi-squared test for differences between fields. Holding all other predictors constant, we found that high-leading teams had an expected shots-on-target rate of 2.239 (*e*^0.806^) times higher than drawing teams, indicating a significant increase of 124%. One-goal-leading teams also showed a significant increase of 79% in their expected shots on target (*p* < 0.001). Table 2 shows an increase in the number of free kicks by 4.8 and 9.8% for high-leading and one-goal-leading teams, respectively. This increase is statistically significant with *p*-values <0.05 and < 0.001, respectively. Conversely, there is a decrease in the number of blocked shots by 12.5 and 12.3% for high-leading and one-goal-leading teams, respectively, and in the number of corner kicks by 12.6 and 12.3%, respectively.

**Table 1:** Estimation results for the Poisson GLM (*N* = 9,256).

Parameters	ShotOn			ShotOff			ShotBlocked		
	*β* (95% CI)	SE	*Z*	*β* (95% CI)	SE	*Z*	*β* (95% CI)	SE	*Z*
Intercept	−1.315 (−1.568, −1.063)	0.129	−10.223^***^	−0.803 (−1.028, −0.577)	0.115	−6.981^***^	−1.907 (−2.219, −1.594)	0.159	−11.967^***^
State:Level^#^ Front2 > Front1 > Level = Behind2 = Behind1				Behind2 > Behind1 > Front1 = Front2			Behind1 = Behind2 > Front1 = Front2		
State:Behind1	−0.044 (−0.108,0.02)	0.032	−1.358	0.155 (0.103, 0.208)	0.027	5.773^***^	0.23 (0.165,0.295)	0.033	6.906^***^
State:Behind2	−0.013 (−0.097, 0.071)	0.043	−0.3	0.294 (0.225, 0.363)	0.035	8.387^***^	0.146 (0.056,0.236)	0.046	3.17^**^
State:Front1	0.582 (0.528, 0.636)	0.028	21.155^***^	−0.075(−0.132, −0.018)	0.029	−2.561^*^	−0.131 (−0.209, −0.054)	0.04	−3.322^***^
State:Front2	0.806 (0.741, 0.871)	0.033	24.366^***^	0.037 (−0.041, 0.115)	0.04	0.928	−0.134 (−0.24, −0.028)	0.054	−2.474^*^
Venue:Away^#^									
Venue:Home	0.136 (0.102, 0.169)	0.017	8.016^***^	0.256 (0.225, 0.287)	0.016	16.115^***^	0.253 (0.212, 0.293)	0.021	12.155^***^
Salary-ratio	0.05 (0.04, 0.061)	0.005	9.595^***^	0.049 (0.04, 0.058)	0.005	10.82^***^	0.057 (0.046, 0.069)	0.006	10.131^***^
Elapsed time	0.00033 (0.00032, 0.00034)	5.4 × 10^−6^	61.559^***^	0.00043 (0.00042, 0.00044)	5.1 × 10^−6^	85.389^***^	0.00043 (0.00041, 0.00044)	6.7 × 10^−6^	63.965^***^
LAttendance	0.116 (0.06, 0.172)	0.029	4.054^***^	−0.022 (−0.071, 0.028)	0.025	−0.846	0.112 (0.043, 0.182)	0.035	3.181^**^
Behind1 × Salary-ratio	0.008 (−0.018, 0.033)	0.013	0.588	−0.008 (−0.03, 0.015)	0.011	−0.665	0.018 (−0.006, 0.042)	0.012	1.49
Behind2 × Salary-ratio	0.012 (−0.032, 0.056)	0.023	0.534	−0.028 (−0.071, 0.014)	0.022	−1.312	0.019 (−0.027, 0.065)	0.024	0.803
Front1 × Salary-ratio	−0.024 (−0.039, −0.008)	0.008	−2.969**	−0.003 (−0.019, 0.013)	0.008	−0.405	−0.02 (−0.041, 0.002)	0.011	−1.804
Front2 × Salary-ratio	−0.016 (−0.031, −0.001)	0.008	−2.065*	−0.031 (−0.048, −0.013)	0.009	−3.409^***^	−0.016 (−0.037, 0.006)	0.011	−1.392
ϕ	0.993			1.170			1.227		
AIC	26751.044			28395.108			23196.691		
pseudo-*R*^2^	0.359			0.412			0.283		

***, **, and * denotes statistical significance at the 0.1, 1, and 5% levels, respectively. *β* denotes estimated coefficients. ^#^Reference categories. ϕ denotes dispersion parameters.

**Table 2:** Estimation results for the Poisson GLM (*N* = 9,256).

Parameters	Freekick			Cornerkick		
	*β* (95% CI)	SE	*Z*	*β* (95% CI)	SE	*Z*
Intercept	0.966 (0.846, 1.087)	0.061	15.747^***^	−0.916 (−1.135, −0.697)	0.112	−8.209^***^
State:Level^#^ Behind1 > Behind2, Behind1 > Front2, Front1 > Front2				Behind2 = Behind1 > Level > Front1 = Front2		
State:Behind1	0.127 (0.097, 0.157)	0.015	8.338^***^	0.222 (0.173,0.27)	0.025	9.024^***^
State:Behind2	0.048 (0.007, 0.088)	0.021	2.308^*^	0.224 (0.16,0.288)	0.033	6.874^***^
State:Front1	0.093 (0.062, 0.124)	0.016	5.916^***^	−0.143 (−0.198, −0.087)	0.028	−5.06^***^
State:Front2	0.047 (0.004, 0.091)	0.022	2.123^*^	−0.135 (−0.212, −0.058)	0.039	−3.421^***^
Venue:Away^#^						
Venue:Home	0.01 (−0.007, 0.027)	0.009	1.163	0.258 (0.229, 0.288)	0.015	17.156^***^
Salary-ratio	0.013 (0.007, 0.019)	0.003	4.396^***^	0.064 (0.056, 0.072)	0.004	16.142^***^
Elapsed time	0.00042 (0.00042, 0.00043)	0.0000028	150.531^***^	0.00042 (0.00041, 0.00043)	0.0000048	87.041^***^
LAttendance	−0.103 (−0.13, −0.077)	0.014	−7.608^***^	0.035 (−0.013, 0.084)	0.025	1.422
Behind1 × Salary-ratio	−0.006 (−0.021, 0.008)	0.007	−0.882	−0.003 (−0.021, 0.016)	0.009	−0.287
Behind2 × Salary-ratio	0.019 (−0.006, 0.044)	0.013	1.503	0.006 (−0.027, 0.039)	0.017	0.364
Front1 × Salary-ratio	−0.007 (−0.017, 0.003)	0.005	−1.401	−0.008 (−0.022, 0.007)	0.007	−1.019
Front2 × Salary-ratio	−0.01 (−0.021, 0.001)	0.006	−1.81	−0.023 (−0.039, −0.007)	0.008	−2.803^**^
ϕ	1.315			1.325		
AIC	42349.377			30476.675		
pseudo-*R*^2^	0.646			0.404		

***, **, and * denotes statistical significance at the 0.1, 1, and 5% levels, respectively. *β* denotes estimated coefficients. ^#^Reference categories. *𝜙* denotes dispersion parameters.

On the other hand, all responses, except for shots on target by trailing teams, were higher than those by drawing teams. The mean shots off target, blocked shots, free kicks, and corners were 34.2, 15.7, 4.9, and 25.1% higher, respectively, for teams trailing by two goals or more than for drawing teams (Tables 1, 2). The mean shots off target, blocked shots, free kicks, and corners were 16.8, 25.9, 13.6, and 24.8% higher, respectively, for one-goal-trailing teams than for drawing teams. All increases were statistically significant (*p* < 0.001).

**Figure 3:**
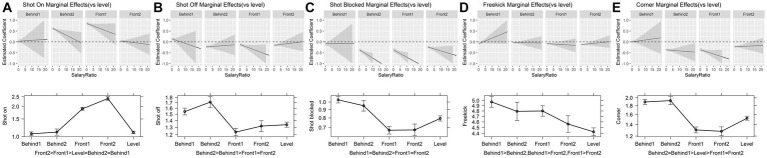
Influence of game state and salary-ratio on the mean number per segment of all the dependent variables.

Moreover, *post hoc* comparisons indicated that leading teams attempted significantly more shots on target per segment than trailing and drawing teams (all *p* < 0.001, Figure 3). Conversely, there were fewer shots off target, blocked shots, and corner kicks when a team led the match. Similarly, one-goal-trailing teams scored more free kicks than high-leading teams (*p* < 0.001).

### 3.2. Heterogeneity effect

There was a significant positive association between the team’s quality and shots on target (***χ***^2^ = 9.595, *e^β^* = 1.052 [1.041–1.063], *p* < 0.001), shots off target (***χ***^2^ = 10.82, *e^β^* = 1.05 [1.041–1.06], *p* < 0.001), blocked shots (***χ***^2^ = 10.131, *e^β^* = 1.059 [1.047–1.071], *p* < 0.001), free kicks (***χ***^2^ = 4.396, *e^β^* = 1.013 [1.007–1.019], *p* < 0.001), and corner kicks (***χ***^2^ = 16.142, *e^β^* = 1.066 [1.058–1.075], *p* < 0.001), whereby an increment of 1 unit (indicating a better quality of the team) was associated with an increase in the mean number of shots on target, shots off target, blocked shots, free kicks, and corner kicks by 5.2, 5, 5.9, 1.3, and 6.6%, respectively.

### 3.3. Game state × salary ratio

Tables 1, 2 show the interaction terms between the heterogeneity effect variables and each performance variable. Notably, significant interactions were observed between the game state and salary ratio of the mean number per segment for shots on target, shots off target, and corner kicks (see Tables 1, 2). However, no significant interaction effects were observed for blocked shots or free kicks. For every one-unit increase in the salary ratio, the mean number per segment for shots on target changed by 1.035 (*e*^0.05–0.016^) for high-leading teams (leading by two or more goals), compared with only 1.052 (*e*^0.05^) for drawing teams. Furthermore, the increment in shots on target in the leading teams was 1.7% lower than that in the drawing teams (*p* < 0.001, Figure 3A) when the salary ratio increased by one unit. Similarly, for shots off target and corner kicks, this drop was 3.2 and 2.4%, respectively (*p* < 0.001; Figures 3B,E).

### 3.4. Venue effect

Based on the GLM, shown in Tables 1, 2, the home advantage was remarkable for the average number of shots on target (***χ***^2^ = 8.016, *e^β^* = 1.145 [1.108–1.184], *p* < 0.001), shots off target (***χ***^2^ = 16.115, *e^β^* = 1.291 [1.252–1.332], *p* < 0.001), blocked shots (***χ***^2^ = 12.155, *e^β^* = 1.287 [1.236–1.341], *p* < 0.001), and corner kicks (***χ***^2^ = 17.156, *e^β^* = 1.295 [1.257–1.334], *p* < 0.001). The average number of shots on target of home teams was 1.145 (*e*^0.136^) times that of visiting teams, a significant increase of 14.5%. The number of shots off target, blocked shots, and corner kicks increased by 29.1, 28.7, and 29.5%, respectively. The mean number of free kicks by home teams was 1% higher than that by visiting teams; however, this difference was insignificant (*p* = 0.245).

### 3.5. Elapsed time and attendance effect

According to the Wald chi-square test for the coefficients, all the variables were significantly associated with elapsed time (all *p* < 0.001). There is clear evidence of an increase in the number of variables with elapsed time.

According to the GLM, the findings presented in Tables 1, 2 indicate a positive correlation between elapsed time and the average number of occurrences per segment for all the variables. Specifically, for every additional second on the elapsed time scale, there was an increase of 0.033, 0.044, 0.043, 0.042, and 0.042% in shots on target, shots off target, blocked shots, free kicks, and corner kicks, respectively. Although this increase was minimal, it was statistically significant (*p* < 0.001). In addition, live attendance was positively associated with shots on target and blocked shots but negatively associated with free kicks.

## 4. Discussion

This study examined the unique effects of game state and heterogeneity in shaping performance under pressure. Our study involving 9,256 segments of the top five European football leagues across 1,826 games in the 2017–18 season broadens our understanding of performance changes under pressure.

### 4.1. Interaction effect

First, this study shows that interim winners tend to perform worse when confronted with weaker opponents. The premise of this study is that, in asymmetric games, favored teams may play with less intensity when intermediate information suggests an inevitable outcome. This study is the first to examine the influence of the interaction between competition heterogeneity and the interim game state on performance.

A potential explanation is that top performance may decrease due to lower motivation to compete. Therefore, we anticipate that winning favorites will be more willing than losing underdogs to decrease their performance in heterogeneous contests, such as saving strength for the next game or diminishing the risk of injury.

These results are similar to those of Ozbeklik and Smith (2017), who propose that more capable competitors would take fewer risks if they possess more advantages.

Furthermore, for outperforming teams, the lower probability of conducting an offensive against a weaker opponent than a stronger opponent is consistent with a shift in focus to slack-related interests. According to Chen and Miller (2007), slack refers to the resources that exceed current performance levels. When a corporation’s performance surpasses expectations, it is more likely to focus on slack, resulting in a negative correlation between performance and slack (Lehman and Hahn, 2013). Ultimately, a team’s primary objective is to win the game rather than simply outscore the opponent by a large margin.

Research indicates that slack can lead to inefficiency, hinder risk-taking, and negatively affect performance, as suggested by Fama (1980) and Jensen and Meckling (1976). Organizational theory suggests that slack can have a positive impact on enterprise performance. In situations where performance is exceptionally high, the relationship between slack and performance may be positive, whereby risk-taking increases as performance improves (Chen and Miller, 2007).

### 4.2. Loss aversion and risk-taking

Our second finding focuses on the influence of the interim game state on performance. This study shows that competitors tend to take more significant risks (shooting more but scoring less and taking more corner and free kicks) when lagging than when in the lead. This means that sportspeople choose higher-risk strategies when in a negative game state and revert to progressively safer strategies when in a tie. This observation may support the negative relationship between performance and risk-taking, which is stronger for losing teams than for winning teams (Lehman and Hahn, 2013). Players tend to adjust their efforts according to potential losses rather than potential gains, which is in line with the concept of loss aversion (Schneemann and Deutscher, 2017).

Normally, a losing team has little to lose except for a worse goal difference. Thus, a losing team may select riskier strategies to catch up with the leaders. According to some researchers (Brown et al., 1996; Grund and Gürtler, 2005; Genakos and Pagliero, 2012; Grund et al., 2013), players who are ranked lower in a competition tend to take more risks than those who are leading. Our findings are consistent with several previous studies showing that struggling organizations and teams that are currently losing are more prone to adopting riskier strategies than industry leaders or teams that are winning in sports competitions. This suggests that the lagging team may be strongly motivated to take bigger risks. Furthermore, a tie score leads to significantly fewer shots on target than when a team is leading the match, and a high lead tends to increase shots on target compared with those attempted by one-goal-leading teams, further suggesting players’ loss aversion (Tversky and Kahneman, 1979).

Interestingly, the potential gain from risk-taking is minimal. Leading teams tend to outperform (more shots on target) trailing and drawing teams. Leading by more than one goal leads to significantly more shots on target than those achieved by a team leading by one goal. The results indicate that there is no monotonic relationship between the number of shots on target a team takes and goal difference. A high lead tends to improve offensive efficiency, whereas a high deficit and balanced score do not affect offensive efficiency.

From the skill perspective, our observation of the inefficiency (fewer shots on target) of disadvantaged teams is consistent with previous reports on the best discriminatory power for the variable “shots on target.” Castellano et al. (2012) suggested that the number of shots on target is a better indicator of team performance than the total number of shots made.

### 4.3. Simple heterogeneity effect

Third, our observations of the positive and significant impact of the indicators of asymmetric competition show that the greater the disparity in salary relative to opponents, the more offense higher-salary teams take on all variables. It is interesting to note that, on average, close competition decreases performance.

In general, the positive impact of matchup heterogeneity is greater for teams with higher salaries than for those with lower salaries. This finding supports the theory that the stronger the rival, the weaker the player’s performance. In heterogeneous fields, the tournament outcome is relatively clear and contestants reduce their efforts (Bach et al., 2009). However, our findings do not support the so-called contamination hypothesis, which indicates that ex ante favorite teams play significantly less intensely in asymmetric games. Based on these results, the stronger the opponent, the weaker the team performance. Our findings are consistent with those of previous studies (Ehrenberg and Bognanno, 1990; Lallemand et al., 2008; Bach et al., 2009; Sunde, 2009).

### 4.4. Limitations

This study has three limitations. First, it must be taken into account that performance can be influenced by match importance. For example, for teams that have already won a league championship, the decisiveness of the remaining games is not high. Future studies should consider and quantify the impacts of match importance. Second, this study is confined to a relatively short period (one season) for only one sport. Future research should verify the robustness of the current findings by using extended observation periods and examining performance measures in other sports. Third, future research should consider gender differences and investigate how the dynamics of the game differ among female soccer players.

## 5. Conclusion

This study examined how soccer players responded to intermediate information in asymmetric contests, focusing on performance feedback in the European Soccer Leagues. In the innovative approach, we measured performance as the number of offensive tactics undertaken during a segment. The results suggested that the favorite would reduce offensive intensity to save costs when intermediate information about the score indicated that an asymmetric game had already been decided. Compared with a tied score, attack intensity was higher for lagging teams and lower for leading teams. In line with risk-taking and loss aversion, players attach more value to potential losses than gains. The empirical results stress that the larger the heterogeneity between the favorite and the underdog, the better the performance of the favorite. The significance of this study lies in enhancing strategic decision making, improving performance analysis, understanding player motivation, and advancing research in professional football. This knowledge can help teams identify areas of improvement and adjust their tactics accordingly. In addition, understanding the strategic decision-making process can provide valuable information on how players’ motivations are influenced by tactical considerations and team dynamics. By delving into these aspects, this study will help researchers and practitioners gain a deeper understanding of the complex interplay between player motivation and performance in professional football.

## Data availability statement

The original contributions presented in the study are included in the article/supplementary material, further inquiries can be directed to the corresponding author.

## Ethics statement

Ethical review and approval was not required for the study on human participants in accordance with the local legislation and institutional requirements. Written informed consent from the patients/ participants or patients/participants' legal guardian/next of kin was not required to participate in this study in accordance with the national legislation and the institutional requirements.

## Author contributions

QY contributed to study conception and design. QY and YZ performed material preparation, data collection, and analysis. The first draft of the manuscript was written by YZ, and all authors commented on previous versions of the manuscript. All authors contributed to the article and approved the submitted version.

## Footnotes

^1^ https://globalsportssalaries.com/GSSS%202017.pdf
